# Lindbladian Decoherence in Quantum Universal Gates: An Insight Analysis for Digital Noise and Thermalisation

**DOI:** 10.3390/e27111089

**Published:** 2025-10-22

**Authors:** José Carlos Rebón, Francisco Delgado

**Affiliations:** Tecnologico de Monterrey, School of Engineering and Science, Atizapán 52926, Mexico

**Keywords:** universal quantum gates, quantum noise, thermalisation, lindblad master equation, decoherence time, dissipation time, parameterisation

## Abstract

Quantum computing is an emergent field promising the improvement of processing speed in key algorithms by reducing their exponential scaling to polynomial, thus enabling solutions to problems that exceed classical computational capabilities. Gate-based quantum computing is the most common approach but still faces high levels of noise and decoherence. Gates play the role of probability mixers codifying information settled in quantum systems. However, they are deviated from their programmed behaviour due to those decoherent effects as a hidden source modifies the desired probability flux. Their quantification of such unavoidable behaviours becomes crucial for quantum error correction or mitigation. This work presents an approach to decoherence in quantum circuits using the Lindblad master equation to model the impact of noise and thermalisation underlying the ideal programmed behaviour expected for processing gates. The Lindblad approach then provides a comprehensive tool to model both probability fluxes being present in the process, thus regarding the gate and the environment. It analyses the deviation of resulting noisy states from the ideal unitary evolution of some gates considered as universal, setting some operating regimes. Thermalisation considers a radiation bath where gates are immersed as a feasible model of decoherence. Numerical simulations track the information loss as a function of the decay rate magnitude. It also exhibits the minimal impact on decoherence coming from particular quantum states being processed, but a higher impact on the number of qubits being processed by the gate. The methodology provides a unified framework to characterise the processing probability transport in quantum gates, including noise or thermalisation effects.

## 1. Introduction

Quantum computing is an approach that uses properties from quantum mechanics, such as superposition and entanglement, to process information using qubits instead of classical bits. It offers the potential to solve certain problems exponentially faster than classical computers. Furthermore, quantum computing could improve key technologies such as cryptography, machine learning, and artificial intelligence [1].

The gate model in quantum computing is the most widely adopted framework [2]. It resembles a classical digital circuit, except it operates with quantum gates (controlled physical operations) acting on qubits to perform computations by breaking down complex tasks into simpler, standardised steps [3]. For a physical system to reliably implement a gate model, it must meet a set of conditions proposed by the physicist David DiVincenzo in 1996 [4], known as the DiVincenzo criteria. These include the ability to (1) physically realise scalable qubits; (2) initialise them to a known state; (3) maintain coherence long enough to perform computations; (4) implement a universal set of quantum gates; and (5) accurately measure individual qubits. For quantum communication, there are two additional criteria: (6) the ability to convert between stationary and flying qubits and (7) the ability to transmit qubits between distant locations. The gate model and DiVincenzo’s criteria shape the theoretical foundation of quantum computing [5].

A set of quantum gates is called universal if it can approximate any unitary operation to arbitrary precision, analogous to how a small set of classical logic gates can realise any Boolean function [6]. Quantum circuits inherit this universality property, and, in practice, the combination of Hadamard, phase, CNOT, and π/8 gates is widely used as a universal set, as it can generate, at least asymptotically, any unitary transformation [7]. Another example of a universal set of gates includes the Hadamard and $\sigma_{z}^{1/4}$ gates which, together with the CNOT gate, provide a fault-tolerant universal basis for quantum computation [8].

In 2018, John Preskill defined the Noisy Intermediate Scale Quantum (NISQ), where the intermediate scale refers to the size of quantum computers ranging from 50 to a few hundred qubits [9], as a period where noise will significantly constrain what quantum computers can do. Noise in NISQ devices comes mainly from decoherence (including bit-flip and dephasing errors due to electromagnetic and thermal fluctuations), gate imperfections, measurement errors, and qubit crosstalk [10]. Error mitigation methods are used to reduce the impact of errors without complete error correction. Researchers define error limits by measuring coherence times, gate fidelity, and readout error rates, establishing a threshold beyond which quantum circuits can no longer produce reliable results [11].

Noise in communication channels, modelled through processes such as bit-flipping, depolarising, and amplitude-damping channels, has helped to understand and mitigate decoherence. Researchers have also applied these models to quantum circuits to simulate the types of noise that impact gate behaviour. The concept of “noise gates” was introduced as a framework in which standard channel noise processes are represented by stochastic operations applied between quantum gates, allowing simulation of decoherence effects in circuits and effectively bridging the gap between channel noise and gate-level noise modelling [12]. Similarly, filter function techniques were used to analyse how time-dependent dephasing noise influences non-trivial gate operations, enabling the design of dynamically corrected gates to suppress specific frequency components of noise [13]. With regard to temperature-dependent noise sources, fault-tolerant pulse sequences have been designed to maintain gate fidelity in the presence of low-frequency noise fluctuations and thermal instability in superconducting and spin qubit systems [14,15]. These developments highlight that noise mitigation techniques originally developed for quantum channels may aid in stabilising gate operations in NISQ devices.

In currently available quantum devices, structured or shaped noise can be reached using the Pauli twirling method [16], which transforms general noise into diagonal syndromes of Pauli noise, as we will use in part of our development. Several Quantum Error Mitigation (QEM) procedures can then be implemented using established techniques such as Probabilistic Error Cancellation (PEC) [17], which estimates the noise by applying inverse transformations to stochastically eliminate the noise. In addition, Zero Noise Extrapolation (ZNE) [18] statistically extrapolates the information to the noiseless limit, while Tensor-Network Error Mitigation (TEM) [19,20] leverages the contractions of the tensor network of quantum circuits to estimate expected values with reduced errors. These methods enable partial suppression of decoherence and gate infidelity without the need of full error correction.

Decoherence in quantum systems due to environmental interactions has previously been modelled using the Lindblad Master Equation (LME). A notable early application to quantum communication examines quantum teleportation through noisy channels using an analytic LME to model various decoherence processes and compute the resulting fidelity loss as a function of noise strength and input-state parameters [21]. Subsequent developments expanded Lindblad-based modelling to include finite-temperature effects; for example, a phonon-induced decoherence approach in semiconductor quantum dots uses a generalised Lindblad form derived from Bloch–Redfield theory [22].

More recently, a 2024 study applied the gate-based Lindblad model to IBM Quantum CNOT gates, showing that this approach accurately captures experimental decoherence through effective Markovian relaxation and dephasing error processes during gate operations, which fits observed fidelity decay better than standard depolarising models [23]. Furthermore, in 2025, a protocol for preparing Gibbs states in thermal equilibrium was presented through Lindblad dynamics engineering, which shows robustness against both temperature and stochastic noise under the hypothesis of eigenstate thermalisation [24]. Another 2025 study presented an explicit analysis of multi-qubit systems interacting with a thermal reservoir, incorporating both spontaneous emission and absorption processes governed by the Bose-Einstein distribution, and developed a fully temperature-dependent Lindblad model for quantum decoherence [25]. Moreover, contemporary research has employed the time-dependent Bloch–Redfield master equation to investigate the impact of environmental interactions on driven qubit dynamics, allowing for more accurate modelling of non-Markovian effects and improving estimates of dephasing rates, gate fidelity, and temperature-dependent decoherence during active quantum control [26]. Together, these studies reinforce the role of the Lindblad equation as a fundamental and adaptable framework for modelling, managing, and mitigating decoherence in quantum information processing under both digital noise and thermal effects.

This work examines whether an LME-based framework can quantify the decoherence from noise and thermalisation by analysing their effects on a universal set of quantum gates. The rest of the article is structured as follows. Section 2 introduces the Hamiltonian and derived prescriptions for the universal gate models to be used; Section 3 presents the LME for noise modelling by quantifying uniform and particular decay rates of noisy effects on the overall quantum states able to be processed; Section 4 similarly describes in this case the thermalisation of universal quantum gates within this context; and Section 5 discusses how this LME-based approach relates to current technologies for quantum computation. Conclusions are settled in the last section.

## 2. Hamiltonian Models to Generate Universal Gates Under Decoherence

This section depicts the interactions and their prescription to reach a set of universal sets of operations or gates under ideal quantum operations. Despite the diversity of possible quantum systems, we set a typical model to ideally reach them before analysing the central aspect in this work, the decoherence of such ideal schemes to quantify the strength of deviation.

Several quantum systems are proposed to perform quantum processing. The related Hamiltonians regard certain mathematical similarities. Thus, comparable Hamiltonians have been employed across a range of physical systems beyond those governed by magnetic fields. For example, in the context of analytically solvable two-level systems, where a driven qubit is subject to single-axis control, the Hamiltonian takes the form $H\left(t\right)=A\left(t\right)\sigma_{x}$, or otherwise a linear combination of $\sigma_{y}$ and $\sigma_{z}$ [27] as a function of the selected axis. This framework treats the drive amplitude A(t) as an arbitrary real-valued function, while the rotation axis remains constant.

Nevertheless, a more controlled Hamiltonian is provided for periodically driven two-level systems; both closed- and open-system Floquet approaches can often be written as an effective Hamiltonian of the following form:

(1) $$\begin{array}{ccc} H(t) & = & H_{0}+\Omega_{0}\sum_{m}f_{m}\left(t\right){\hat{n}}^{m}\cdot {\vec{\sigma }}^{m} \end{array}$$

where the drive amplitude varies in time while the rotation axis remains fixed, and ${\vec{\sigma }}^{m}=\left(\sigma_{x}^{m},\sigma_{y}^{m},\sigma_{z}^{m}\right)$ [28]. In the high-frequency regime, analysis of driven spins shows that the stroboscopic generator preserves this fixed-axis form with a renormalised amplitude [28], and comparable results emerge from open-system Floquet treatments of LMEs [29].

Although the Hamiltonians describing photonic quantum systems are not always as directly expressed in Pauli form as those for spin systems in magnetic fields, similar analytical approaches have been employed. In polarisation-encoded photonic qubits, unitary evolutions such as polarisation rotations are readily represented as single-qubit operations in the Pauli basis [30]. Entangled photon pairs, generated via spontaneous parametric down-conversion, provide two-qubit states whose transformations, including controlled operations, can be decomposed into tensor products of Pauli operators [31,32]. More generally, in photonic quantum information processing (including linear optical quantum computing), optical components used in experiments are typically described by breaking down the corresponding quantum gates into combinations of Pauli operators [33].

### 2.1. Generation of Single-Qubit Universal Gates and Prescriptions

In this section, we will focus on local gate operations. Although a large number of possible quantum systems can establish quantum processing, we will consider the following Hamiltonian:

(2) $$\begin{array}{ccc} H(t) & = & -\frac{\hbar }{2}\Omega \left(t\right)\hat{n}\cdot \vec{\sigma } \end{array}$$

which, in fact, is likely similar to magnetic systems but anyway sets a linear combination of Pauli operators. Still, in such a case, Ω(t) barely represents the local magnetic field on the single spin system. Note that despite the time dependence, the direction, in terms of the linear combination of Pauli operators, is fixed through the unitary vector $\hat{n}$.

For photonic systems, Hamiltonian modelling could be simpler because there are no electromagnetic induction effects, providing an easier construction of gates. However, in this case, we will consider a smooth pulse envelope in the form of a Wideband, Uniform Rate, Smooth Truncation (WURST) more useful for the electromagnetic case. The wide bandwidth of this envelope allows it to cover a wider range of frequencies; its uniform rate allows uniformly distributed energy over time, and smooth truncation avoids discontinuities [34]. WURST pulses are often used as envelopes for periodic pulses with variable chirp frequencies [35]. The underlying principles of WURST pulses have shown promise in hybrid quantum systems involving superconducting coplanar waveguides, in particular, for electron spin control in potential quantum memories applications [36]. WURST-type pulses are important as they can handle imperfections such as variations in frequency or signal strength [37]. Furthermore, chirped and polychromatic pulses have also been explored within superconducting qubit systems: a 2022 study showed that using a set of pulses at different frequencies (called polychromatic pulse trains, or PPTs) can reliably control IBM’s superconducting qubits while offering robustness to detuning errors and gate imperfections [38]. The WURST pulse in this work is characterised by the following definitions:

(3) $$\begin{array}{ccc} \Omega (t) & = & \Omega_{0}\theta_{0}\left(t\right) \end{array}$$

(4) $$\begin{array}{ccc} \theta_{0}\left(t\right) & = & W(t)\sin(t\;\omega (t)) \end{array}$$

(5) $$\begin{array}{ccc} W(t) & = & 1-{\left|\cos\left(\pi t\right)\right|}^{n} \end{array}$$

(6) $$\begin{array}{ccc} \omega (t) & = & 4\alpha \left(1-t\right)+\omega_{0} \end{array}$$

**Figure 1:**
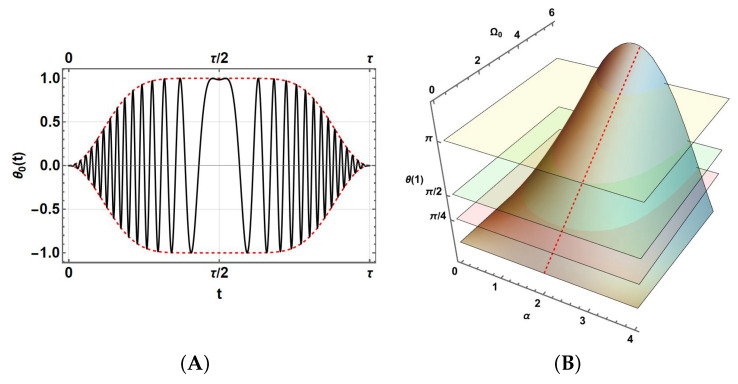
(**A**) Pulse $\theta_{0}$ (black line) with its envelope W(t) (dashed red line) and a variable frequency chirp during its period τ=1. (**B**) Integral θ(1) for the pulse Ω(t) during an entire period as a function of α and $\Omega_{0}\left(t\right)$, showing the relevant solutions $\theta \left(1\right)=\frac{\pi }{4},\frac{\pi }{2}$, and π.

$\Omega_{0}$ is the amplitude, while $\theta_{0}\left(t\right)$ is a combined signal built by the envelope W(t) and a sine oscillatory function depending on a linear variable frequency ω(t) with a variable decreasing rate 4α. The smoothness of W(t) depends on the lowest integers *n*. Figure 1A shows a typical WURST signal, illustrated for $\Omega_{0}=1,\omega_{0}=0,n=8$ and constructed over a rescaled unitary period τ=1 (this aspect will be discussed later in this section). Thus, it is ideally introduced in the Schrödinger equation for the evolution operator U(t):

(7) $$\begin{array}{c} H\left(t\right)U\left(t\right)=i\hbar \frac{\partial U(t)}{\partial t} \end{array}$$

Then, the evolution operator becomes analytical and is obtained as follows:

(8) $$\begin{array}{ccc} U(t) & = & e^{-\frac{i}{\hbar }\int_{0}^{t}H\left(t^{\prime }\right)dt^{\prime }}=\sigma_{0}\cos\frac{\theta (t)}{2}+i\hat{n}\cdot \vec{\sigma }\sin\frac{\theta (t)}{2}\equiv R_{s}^{\hat{n}}\left(\theta \left(t\right)\right)\\ & & \mathrm{with}:\;\theta \left(t\right)=\int_{0}^{t}\Omega \left(t^{\prime }\right)dt^{\prime } \end{array}$$

a rotation on the subsystem *s*, around the axis $\hat{n}$ by an angle θ(t). In the following, we state the concrete prescriptions for reproducing the local universal gates $U_{\pi /4},U_{\pi /8},H$. In fact, in terms of Equation (8), the gate $U_{\pi /4}$ is obtained with the following:

(9) $$\begin{array}{ccc} U_{\pi /4} & = & \left(\begin{array}{cc} 1 & 0\\ 0 & e^{i\frac{\pi }{2}} \end{array}\right)≃\left(\begin{array}{cc} e^{-i\frac{\pi }{4}} & 0\\ 0 & e^{i\frac{\pi }{4}} \end{array}\right)=\sigma_{0}\cos\frac{\pi }{4}-i\sigma_{z}\sin\frac{\pi }{4}\\ & & \mathrm{then}:\;\theta \left(1\right)=\frac{\pi }{2},\;\hat{n}=-\hat{k} \end{array}$$

while the $U_{\pi /8}$ gate is reached if the following restriction is met

(10) $$\begin{array}{ccc} U_{\pi /8} & = & \left(\begin{array}{cc} 1 & 0\\ 0 & e^{i\frac{\pi }{4}} \end{array}\right)≃\left(\begin{array}{cc} e^{-i\frac{\pi }{8}} & 0\\ 0 & e^{i\frac{\pi }{8}} \end{array}\right)=\sigma_{0}\cos\frac{\pi }{8}-i\sigma_{z}\sin\frac{\pi }{8}\\ & & \mathrm{then}:\;\theta \left(1\right)=\frac{\pi }{4},\;\hat{n}=-\hat{k} \end{array}$$

Finally, the Hadamard gate is obtained for the following:

(11) H=121−11−1=σ1+σ32≃σ0cosπ2+i2(σx+σz)sinπ2then:θ(1)=π,n^=12(ı^+k^)

Thus, these prescriptions, in addition to the correct selection of $\hat{n}$, depend on the correct selection of $\Omega_{0},\omega_{0},\alpha$. For the selection of $\omega_{0}=0$, Figure 1B shows θ(τ=1) for a set of values of these parameters. Horizontal planes establish the necessary restrictions settled by Equations (9)–(11). Then, there are many possible prescriptions. In the following, we will choose the lowest $\Omega_{0}$ values by setting α=2 in the case with $\omega_{0}=0$ (on the dashed red curve on the surface). They can be achieved numerically, becoming ${\Omega_{0}}_{U_{\pi /4}}=2.32967,{\Omega_{0}}_{U_{\pi /8}}=1.16484,{\Omega_{0}}_{U_{H}}=4.65935$, which are in fact linear.

### 2.2. Generation of a CNOT Gate and Prescriptions

To reach an entangling gate, such as CNOT, which is part of the universal set, we require an interaction between two qubits. For example, the Ising–Heisenberg interaction can be employed [39,40], which could still be complemented by local operations such as those described above. In this case, we will consider the following interaction:

(12) $$\begin{array}{ccc} H(t) & = & \sum_{i,j=1}^{3}J_{ij}\sigma_{i}^{1}⊗\sigma_{j}^{2}-\sum_{i=1}^{2}\frac{\hbar }{2}\Omega_{i}\left(t\right){\hat{n}}^{i}\cdot {\vec{\sigma }}^{i} \end{array}$$

with $J_{ij}$, the strengths of the two-qubit interactions, with complementary local operations of strength $\frac{\hbar }{2}\Omega_{i}\left(t\right)$ for each qubit.

However, in the current analysis we use a well-known decomposition of the CNOT gate, the Cartan decomposition [41]. It is implemented using local rotations on several axes as in Equation (8), together with a dipole–dipole Ising-like interaction aligned to the *z*-axis and free of local rotations, $H\left(t\right)=-\hbar J\sigma_{3}^{1}⊗\sigma_{3}^{2}$. Its single form provides a simple evolution operator:

(13) $$\begin{array}{ccc} U(t) & = & \cos\phi \left(t\right)\;\sigma_{0}^{1}⊗\sigma_{0}^{2}+i\sin\phi \left(t\right)\;\sigma_{3}^{1}⊗\sigma_{3}^{2}\equiv A\left(\phi \left(t\right)\right) \end{array}$$

assuming that J is a constant, then ϕ(t)=Jt; otherwise, the previous argument should be easily adapted to $\phi \left(t\right)=\int_{0}^{t}J\left(t\right)dt$. Then, the Cartan decomposition of the CNOT gate is as follows:

(14) $$\begin{array}{ccc} C^{1}NOT_{2} & = & e^{i\frac{\pi }{4}}R_{1}^{-\hat{k}}\left(\frac{\pi }{2}\right)R_{2}^{\hat{j}}\left(\frac{\pi }{2}\right)R_{2}^{\hat{k}}\left(\frac{\pi }{2}\right)A\left(\frac{\pi }{4}\right)R_{2}^{-\hat{j}}\left(\frac{\pi }{2}\right) \end{array}$$

In the following, we will skip the unphysical factor $e^{i\frac{\pi }{4}}$. Rotations have similar prescriptions as those provided in Equation (9).

## 3. Lindblad Equation to Add Noise Models Disturbing the Gates

To model the effect of decoherence on the gates, we will consider the Lindblad equation, immersing the Hamiltonian models and prescriptions developed above:

(15) $$\begin{array}{ccc} \frac{\partial \rho }{\partial t} & = & -\frac{i}{\hbar }\left[H,\rho \right]+L_{B} \end{array}$$

(16) $$\begin{array}{ccc} & & \mathrm{with}:\;L_{B}=\sum_{i=1}^{n}\gamma_{i}\left(L_{i}\rho L_{i}^{†}-\frac{1}{2}\left\{L_{i}^{†}L_{i},\rho \right\}\right) \end{array}$$

For the time-dependent Hamiltonians considered, this equation could be solved numerically for the density matrix ρ under the ideal parameters that generate each previous universal gate to quantify decoherence in the appropriate models for Lindblad ladder operators $\left\{L_{i}\right\}$, which represent the system-environment interactions. The $\gamma_{i}$ are non-negative decay rates, corresponding to dissipative processes. Negative values of $\gamma_{i}$ can occur in certain extended models such as those derived from the Bloch–Redfield equation, but these may lead to non-physical dynamics and violate the positivity of the density matrix [42,43].

In the previous section, it was assumed that each single-bit gate was performed in a unit time τ=1. Using τ for such a scaled time, in fact, if each gate is achieved in a characteristic time $t=T_{G}$, this time scaling $t\to \tau =\frac{t}{T_{G}}$ produces a rescaling in $\Omega_{0}\to \Omega_{0}T_{G}$ and in the Lindblad coefficients $\gamma_{i}\to \gamma_{i}T_{G}$. It clearly implies that if $T_{G}$ becomes shorter, then the amplitude $\Omega_{0}$ must be reduced to carry out the gate together with the $\gamma_{i}$ amplitudes in the new time scale.

Lindblad decay rates $\gamma_{i}$ encountered in current applications are most often reported in the kHz–MHz range for solid-state qubits. However, there are notable cases in the literature in which significantly smaller values are considered. For example, in a study of four-qubit entanglement dynamics, decoherence was modelled with a Lindblad rate of $\gamma =0.01\;s^{-1}$, which led to an increase in entanglement after tens of seconds [25]. Experimental Lindblad tomography of superconducting qubits reports decay rates in the range of 0.001–$0.004\;s^{-1}$, consistent with coherence times in the submillisecond regime [44].

When mapped to physical timescales set by the Rabi frequency, which is in the kHz to MHz range, these correspond to effective rates in the range $10^{6}$ to $10^{9}$ $s^{-1}$. Current quantum computer technologies use transmon-based superconducting circuits [45], which, although they can be associated with effective electric dipole interactions, are ruled by different Hamiltonians than Equations (2) and (12). As we shall see in the final discussion section, it moves characteristic gate operation times around $T_{G}\approx 10^{-5}–10^{-6}$ s and $\gamma_{i}$ decay rates to ∼$10^{4}$–$10^{6}$ $s^{-1}$. Motivated by these considerations, in our study we used decay rates $\gamma_{i}$ spanning 0.001 to 1 $s^{-1}$ in terms of a unitary dimensionless time (precisely transformed by factor $T_{G}$).

### 3.1. Trace Distance: A Measure to Track the Impact of Noise on Ideal Gates

The difference between the ideal effect of the gates on the specific states of the qubit, written in the density matrix $\rho_{id}$, could be obtained with respect to the real state derived from the Lindbladian approach ρ. This quantification can be achieved by means of the trace distance:

(17) $$\begin{array}{ccc} \Delta & = & \frac{1}{2}\mathrm{Tr}\left[\sqrt{\left(\rho -\rho_{id}\right){\left(\rho -\rho_{id}\right)}^{†}}\right]\in \left[0,1\right] \end{array}$$

Although other alternative measures could be used to compare output states (for instance, fidelity, Bures distance, Hilbert–Schmidt distance), they are generally not distance monotones, thus providing different measurement scales but still capturing the difference between matrices. The trace distance between two quantum states, represented by the density matrices ρ and σ, is defined as half the trace norm of their difference, $D\left(\rho ,\sigma \right)=\frac{1}{2}\mathrm{Tr}\left|\rho -\sigma \right|$. This metric quantifies the distinguishability of quantum states, becoming valuable for measuring the study of decoherence in open quantum systems by putting emphasis on density matrix populations, the diagonal elements of ρ. It ranges from zero (identical states) to one (states are perfectly distinguishable; i.e., their supports are orthogonal) [46]. In addition, it is the quantum generalisation of the Kolmogorov distance for classical probability distributions, as is the density matrix. In any case, the use of this or another measure detects identical matrices and sets a scale to compare differences between matrices. Values strictly between zero and one occur when the states are different but not necessarily orthogonal, i.e., they have some overlap in their support. Key properties of trace distance include non-negativity, symmetry, triangle inequality, unitary invariance, contractivity under completely positive trace-preserving maps, and convexity, which collectively establish it as a reliable measure in quantum information theory [7]. However, it exhibits certain limitations such as non-monotonicity under tensor products and computational complexity when applied to high-dimensional systems [47]. Despite these restrictions, trace distance remains fundamental for tasks such as quantum discrimination and error correction [46,47].

In our analysis, we will compare as a limit case a density matrix ρ representing a total depolarising mixed state; it will appear as an asymptotic behaviour when $\gamma_{i}\to \infty ,i=1,\ldots ,n$. For *N* qubits, they will become $\rho_{ii}=2^{-N}$. Instead, it will be compared to σ, a density matrix for a pure state obtained for $\gamma_{i}=0,i=1,\ldots ,n$. This matrix will exhibit a well-defined quantum state with a certain unique population $\rho_{kk}$ filled; that is, $\rho_{ii}=\delta_{k,i}$. Thus, $\Delta \to \frac{1}{2}\left(\left(2^{N}-1\right)2^{-N}+\left(1-2^{-N}\right)\right)=1-\frac{1}{2^{N}}$ as the limit case for the highest and noisiest decoherence, $\frac{1}{2}$ and $\frac{3}{4}$ for N=1 and N=2 qubits, respectively.

### 3.2. A Single Model of Non-Coherent Effects Generating Noise on Qubits Gates

In the following, we analyse the noise introduced in single local gates considering $L_{i}=\sigma_{i}$, the sparse Pauli–Lindblad (PL) noise model [16,48]. Although a general representation is given by $L_{ij}=\sum_{j=0}^{3}l_{ij}\sigma_{j}$, this simplified model represents emblematic noise mainly characterised as bit-flipping and dephasing noise.

(18) $$\begin{array}{ccc} L_{B_{\mathrm{noise}}^{1}} & = & \sum_{i=1}^{3}\gamma_{i}\left(\sigma_{i}\rho \sigma_{i}^{†}-\rho \right) \end{array}$$

An important feature of this model is that the average gate fidelity [49] for its associated Pauli channel depends on $\sum_{i=1}^{3}\gamma_{i}$. In addition, when $\gamma_{1}=\gamma_{2}=\gamma_{3}$, this model becomes invariant under an orthonormal transformation in the set $\left\{\sigma_{i}|i=1,2,3\right\}$ (see Appendix A).

#### 3.2.1. Uniform Noise on Local Gates

**Figure 2:**
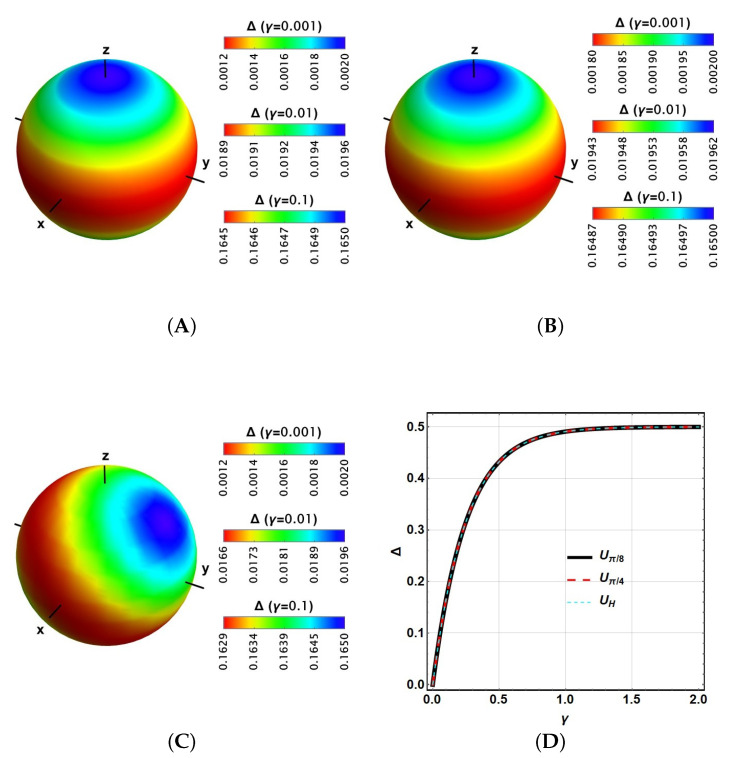
Δ for the pure states on the Bloch sphere for (**A**) π/4 gate, (**B**) π/8 gate, and (**C**) Hadamard gate, considering γ=0.001,0.01and0.1. (**D**) Plot of average Δ as a function of γ for the three previous gates.

Since both ρ and Δ depend on the state $\rho_{0}=|\psi_{0}\rangle \langle \psi_{0}|$ that is processed by the gate, the results for Δ should be represented as functions of this input state $|\psi_{0}\rangle =\cos\frac{\theta }{2}|0\rangle +\sin\frac{\theta }{2}e^{i\phi }|1\rangle$ parameterised on the Bloch sphere θ,ϕ. Our first analysis will consider the same contribution for each type of noise, $\gamma_{i}=\gamma$. The results are graphically represented in Figure 2.

Figure 2A shows each outcome for Δ in the Bloch sphere for $U_{\pi /4}$, referring to each input state $\rho_{0}$. Δ is reported in colour, in agreement with the colour legends on the right. The axes are labelled x,y,z as for spin systems, but their positive directions correspond to $\left|+\rangle ,\right|Y_{+}\rangle ,|0\rangle$ states (with |±〉 and $|Y_{\pm }\rangle$, the eigenstates of $\sigma_{x}$ and $\sigma_{y}$ respectively, with eigenvalues ±1), respectively. Although for different values of γ, the plots exhibit the same structure but on a different scale, so different legends are included for different values of this parameter. It is noticed that there are tiny variations with higher values for Δ as expected, but with a narrower relative range as γ increases. Then, in a certain sense, decoherence is almost insensible from $\rho_{0}$. The same behaviour is observed for $U_{\pi /8}$ in Figure 2B with still narrower ranges. A similar behaviour is observed in Figure 2C for the Hadamard gate. Finally, Figure 2D reports a mean value on the Bloch sphere for Δ as a function of γ, exhibiting identical asymptotic behaviour at Δ=0.5 when γ increases, independently from the gate.

The relation between fidelity F (a common average quantity reported in the performance of gates for several technologies) and Δ is in order, then with the values γ. Due to the Fuchs-van de Graaf inequality [50], there is a clear relation between the first two quantities: ${\left(1-\Delta \right)}^{2}\leq F\leq 1-\Delta^{2}$. Note that these values become opposite; while Δ decreases, then F increases. The approximate corresponding values of Δ for different values of γ are barely double (see Figure 2): Δ≈0.2 (γ=0.1),Δ≈0.02 (γ=0.01),Δ≈0.002 (γ=0.001). Then, the corresponding bounding values for F become 0.6400–0.9600,0.9604–0.9996,0.9960–0.9999, respectively.

#### 3.2.2. Differentiating the Strengths of Noise on Qubits Gates

This section presents an analysis of the case where the strength coefficients $\gamma_{i}$ become different, thus promoting more of some noisy effects than others. Parameterising those three coefficients in terms of the new parameters θ, ϕ, and $\gamma_{\max}$:

(19) $$\begin{array}{c} \left(\gamma_{1},\gamma_{2},\gamma_{3}\right)=\gamma_{\max}\left(\sin^{2}\theta \cos^{2}\phi ,\sin^{2}\theta \sin^{2}\phi ,\cos^{2}\theta \right),\;\mathrm{with}:\;\gamma_{i}\geq 0,i=1,2,3 \end{array}$$

we can analyse again the previous problem, thus finding important differences. Note that this representation is feasible because each coefficient $\gamma_{i}$ appears as $\sqrt{\gamma_{i}}$ together with each Lindblad operator $\sigma_{i}$ in the LME. However, we have many parameters because the outcome depends not only on the initial state processed by the gate but also on those values $\gamma_{i}$. For that reason, in this case, we have used the Monte Carlo method on the Bloch sphere to find the maximum Δ there. Then, we report max(Δ) as a function of the spherical representation θ,ϕ, for some values $\gamma_{\max}$.

**Figure 3:**
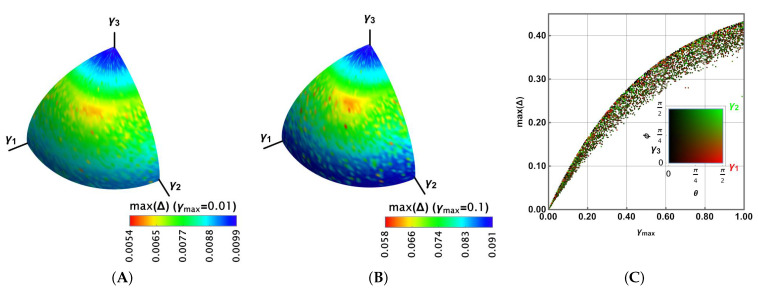
Maximal Δ on the Bloch sphere for the π/4 gate in colour, in agreement with the bottom legend for the group of parameters $\gamma_{i},i=1,2,3$ for (**A**) $\gamma_{\max}=0.01$, and (**B**) $\gamma_{\max}=0.1$. (**C**) Maximal Δ as a function of $\gamma_{\max}$ for a set of $10^{4}$ random sets of $\gamma_{i},i=1,2,3$ colours in agreement with their spherical representation readable in the legend.

Figure 3 reports the results for $U_{\pi /4}$. Because $\gamma_{i}\geq 0$ to preserve the positivity and trace of ρ, the outcomes are reported only on the positive octant. Figure 3A shows the results max(Δ) for $\gamma_{\max}=0.01$ in colour. A broader range is obtained compared to the previous case. Moreover, in Figure 3B, the case $\gamma_{\max}=0.1$ is shown. It shows a larger region for the highest values of max(Δ) and a wider range; thus, the distribution is biased toward the larger values. Figure 3C shows $10^{4}$ random values for $\gamma_{\max}\in \left[0,1\right]$ and for $\theta \in \left[0,\frac{\pi }{2}\right],\phi \in \left[0,\frac{\pi }{2}\right]$. In this case, max(Δ) shows a distribution with the same average behaviour as γ in Figure 2D (noting that there is a correspondence $3\gamma ⟷\gamma_{\max}$). In that distribution, each random dot was coloured according to its proximity to the $\gamma_{1},\gamma_{2},\gamma_{3}$ axis, as the legend at the bottom shows (black for $\gamma_{3}$, green for $\gamma_{2}$, and red for $\gamma_{1}$). No tendency is observed in the distribution as a function of that closeness.

**Figure 4:**
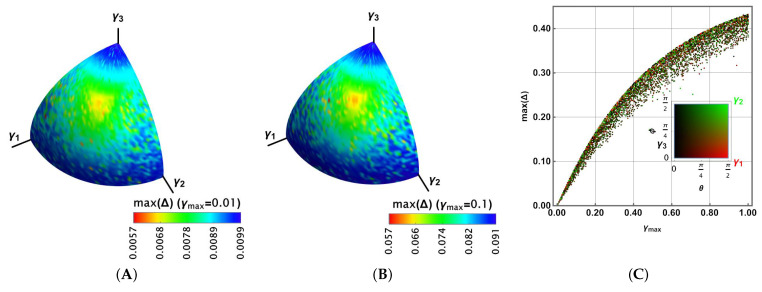
Maximal Δ on the Bloch sphere for the π/8 gate in colour, in agreement with the bottom legend for the group of parameters $\gamma_{i},i=1,2,3$ for (**A**) $\gamma_{\max}=0.01$, and (**B**) $\gamma_{\max}=0.1$. (**C**) Maximal Δ as a function of $\gamma_{\max}$ for a set of $10^{4}$ random sets of $\gamma_{i},i=1,2,3$ colours in agreement with their spherical representation readable in the legend.

For $U_{\pi /8}$, a similar behaviour is observed in Figure 4A,B for $\gamma_{\max}=0.01,0.1$, respectively. However, the highest values of max(Δ) appear to cover more extended regions. It can be seen in Figure 4C, where the distribution is slightly biased towards the larger values of max(Δ), with more dispersed values.

**Figure 5:**
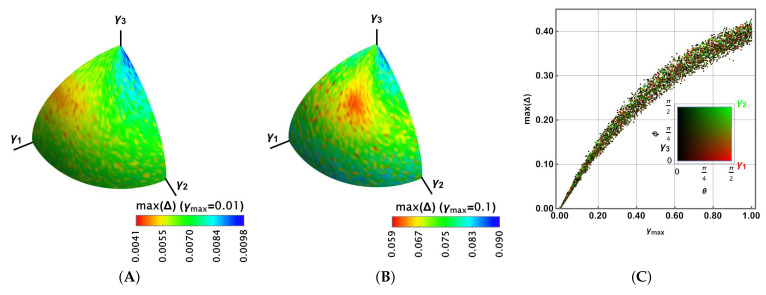
Maximal Δ on the Bloch sphere for the Hadamard gate in colour, in agreement with the bottom legend for the group of parameters $\gamma_{i},i=1,2,3$ for (**A**) $\gamma_{\max}=0.01$, and (**B**) $\gamma_{\max}=0.1$. (**C**) Maximal Δ as a function of $\gamma_{\max}$ for a set of $10^{4}$ random sets of $\gamma_{i},i=1,2,3$ colours in agreement with their spherical representation readable in the legend.

Finally, for the Hadamard gate in Figure 5, plots A and B show a reduced density for the highest values of max(Δ), although they have narrower ranges. In fact, Figure 5C exhibits a more central distribution for the values of max(Δ). In any case, for the three gates, these values are asymptotic to 0.5, reducing their dispersion for the lowest and highest values of $\gamma_{\max}$.

In the further development of this article, we will identify the general Lindblad coefficient G as the coefficient obtained by maintaining individual coefficients $l_{i}$ in the Lindblad operators $L_{i}$ such that $\sum_{i}^{n}{\left|l_{i}\right|}^{2}=1$. Thus, in this case, $G_{\mathrm{noise}}^{1}=\gamma_{\max}$ (alternatively, $G_{\mathrm{noise}}^{1}=3\gamma$ in our initial analysis).

### 3.3. Noise on a CNOT Gate

For two-qubit gates, the Lindblad operators of PL will become $L_{ij}=\sigma_{i}^{1}⊗\sigma_{j}^{2}$, then:

(20) $$\begin{array}{ccc} L_{B_{\mathrm{noise}}^{2}} & = & \sum_{i,j=1}^{3}\gamma_{ij}\left(\sigma_{i}^{1}⊗\sigma_{j}^{2}\rho \sigma_{j}^{1†}⊗\sigma_{i}^{2†}-\rho \right) \end{array}$$

We have already analysed that individual differentiated terms $\gamma_{i}$ just barely add to the overall Δ for one-qubit gates. For that reason and for simplicity, in the current analysis we will use the same value for all types of noise $\gamma_{ij}=\Gamma$ (note that for comparison purposes, in this case $G_{\mathrm{noise}}^{2}\equiv 9\Gamma$ becomes comparable to 3γ and $\gamma_{\max}$ of the previous cases analysed). In addition, we construct a composed Hamiltonian with the five steps for Cartan decomposition in Equation (14):

(21) $$\begin{array}{c} H\left(t\right)=\left\{\begin{array}{cc} +\frac{\hbar }{2}\Omega_{U_{\pi /2}}\left(\tau \right)\sigma_{0}^{1}⊗\sigma_{2}^{2} & ,\;\tau \in [0,1)\\ -\frac{\pi \hbar }{4}\sigma_{3}^{1}⊗\sigma_{3}^{2} & ,\;\tau \in [1,2)\\ -\frac{\hbar }{2}\Omega_{U_{\pi /2}}\left(\tau -2\right)\sigma_{0}^{1}⊗\sigma_{3}^{2} & ,\;\tau \in [2,3)\\ -\frac{\hbar }{2}\Omega_{U_{\pi /2}}\left(\tau -3\right)\sigma_{0}^{1}⊗\sigma_{2}^{2} & ,\;\tau \in [3,4)\\ +\frac{\hbar }{2}\Omega_{U_{\pi /2}}\left(\tau -4\right)\sigma_{3}^{1}⊗\sigma_{0}^{2} & ,\;\tau \in [4,5] \end{array}\right. \end{array}$$

In the current analysis, we skip to provide a continuous Hamiltonian for t∈[1,2], which could be smoothly achieved with a WURST signal, αW(τ) with $\alpha \int_{0}^{1}W\left(\tau \right)d\tau =\frac{\pi }{4}$, instead of the constant being used. Also, for representation purposes, we consider two-qubit pure states with real coefficients, parameterised as follows [51]:

(22) $$\begin{array}{ccc} |\psi \rangle & = & \sin\alpha \sin\beta \cos\gamma |00\rangle +\sin\alpha \sin\beta \sin\gamma |01\rangle +\sin\alpha \cos\beta |11\rangle +\cos\alpha |10\rangle \\ & & \mathrm{with}:\;\alpha ,\beta ,\gamma \in [0,\pi ] \end{array}$$

thus constructing the initial density operator, $\rho_{0}=\left|\psi \rangle \langle \psi \right|$. The concurrence is defined as $C=1-4\det({\mathrm{Tr}}_{k}\rho_{0}),k=1,2$ for the k- partial trace of $\rho_{0}$. It becomes the following:

(23) $$\begin{array}{ccc} C & = & 1-4\sin^{2}\alpha \sin^{2}\beta {\left(\cos\gamma \sin\alpha \cos\beta -\sin\gamma \cos\alpha \right)}^{2} \end{array}$$

**Figure 6:**
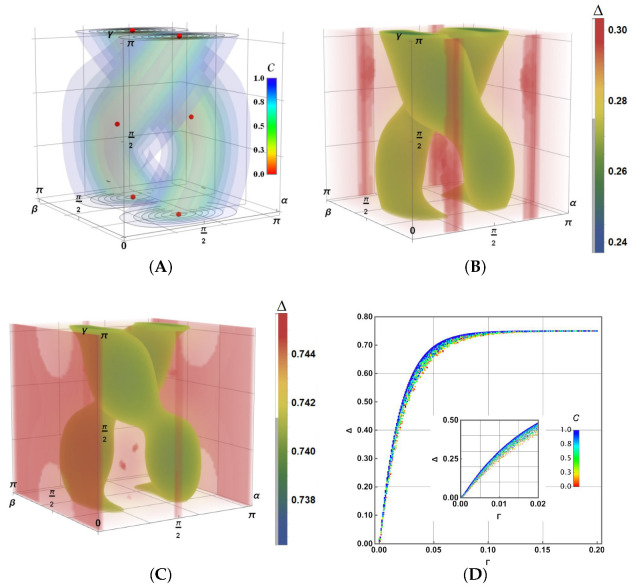
(**A**) Parametric space for the 2-qubit pure states with real coefficients. Double-layer density maps for Δ as a function of α,β,γ corresponding to (**B**) Γ=0.01, and (**C**) Γ=0.1. (**D**) Δ for a sweep of $10^{4}$ random states in the parametric space over Γ coloured in agreement with their concurrence C.

If C=1, the state is separable; instead, if C=0, the state is maximally entangled. Other values in between are partially entangled states. Then we introduce a parametric space [51] to represent our results in Figure 6A, which shows this space characterised by the contour surfaces for C. In this representation, the separable states are located on the lateral faces of the cubic space (α=0,π and β=0,π) and on the surface tanαcosβ=tanγ, all coloured blue in Figure 6A. The maximum entangled states are located in the central helix lines inside the reddest tubes. The red points represent the Bell states.

Using Equations (15), (20) and (21) together, we numerically solve the corresponding Lindblad equation, then obtain Δ for each point of the parametric space in Figure 6A. To represent these outcomes, we employ double-layer density maps for Δ in Figure 6B (for Γ=0.01) and C (for Γ=0.1) as a function of α,β,γ in the parametric state space in agreement with the colour bar. There, solid colours report the values referred to by the grey bars on the left side of the colour legend (note in this case the upper tiny grey bar referring to the maximal values for Δ), whereas transparent colours comprehend the values marked by the left white region of the bar.

In Figure 6B,C, note that the maximum values of Δ correspond to pure states, while the minimum values of Δ correspond to the entangled states. Also note that the measured error increases as Γ increases, although their ranges remain reduced. Moreover, the relative Δ ranges become reduced more when Γ increases; notice the increased strangulation of green regions for Γ=0.1. However, there is no correlation between maximum error and entanglement. Notice that the solid-coloured regions (the minimum values for Δ) do not completely match the helicoidal regions of maximum entanglement. For Γ=0.01, the maximal values of Δ appear for the initial states |1〉⊗|+〉 and |1〉⊗|−〉 ($\alpha =-\frac{\pi }{4},\frac{\pi }{4}$ and β=0,π). Instead, for Γ=0.1, the maximum errors appear for |0〉⊗|1〉 (α=0,π) and |1〉⊗|1〉 ($\alpha =\frac{\pi }{2}$ and β=0,π).

Finally, as before, Figure 6D reports Δ for a sweep of $10^{4}$ random states in the parametric space through different values of Γ. Δ becomes asymptotic to 0.75 when Γ increases. Each initial state was coloured according to C in the legend on the right. Note, as before, that pure states C=1 have the highest error Δ. An important aspect is that Δ becomes less dispersed as Γ increases, as already observed in Figure 6B,C. The inset shows the details of Δ for Γ∈[0,0.2]. In general, we observed a notable increase in Δ compared to the one-qubit gates, a fact experimentally observed [52,53]. The gates processing more than one qubit can be decomposed using a series of single-qubit and CNOT gates [7], the accumulative effect of noise due to this series is also important, an aspect not analysed here. Furthermore, for the corresponding values γ and Γ in Figure 2D and Figure 6D, the value of Δ reaches the asymptotic values more quickly, particularly because the CNOT gate consisted of several gates of one or two qubits. Using the Fuchs-van de Graaf inequality, the bounding values for F for Γ=0.1(Δ≈0.75), 0.01(Δ≈0.25), and 0.001(Δ≈0.03) (from Figure 6) become 0.0625–0.4375, 0.5625–0.9375, and 0.9409–0.9991, respectively.

### 3.4. Some Remarks About PL Noise Model, Damping Noise, and Dephasing Noise

In our development of Section 3.2.1, if we use the energy eigenstates $|\epsilon_{0}\rangle ,|\epsilon_{1}\rangle$ (with $E_{0}<E_{1}$ as their respective eigenvalues) as a basis, instead of those of the spin in *z*-direction (it means that Pauli operators have the same form, but are intended for this basis), if $\gamma_{1}=\gamma_{2}$, we note the following:

(24) $$\begin{array}{c} \sigma_{1}\rho \sigma_{1}^{†}+\sigma_{2}\rho \sigma_{2}^{†}=2\left(\sigma_{-}\rho \sigma_{-}^{†}+\sigma_{+}\rho \sigma_{+}^{†}\right) \end{array}$$

where $\sigma_{-}=|\epsilon_{0}\rangle \langle \epsilon_{1}|=\sigma_{+}^{†}$. Thus, $\sigma_{1}=\sigma_{-}+\sigma_{+}$ and $i\sigma_{2}=\sigma_{-}-\sigma_{+}$. Then, we can write the corresponding Lindblad term ($\gamma_{\mathrm{relax}}=2\gamma_{1}=2\gamma_{2}$) as follows:

(25) $$\begin{array}{ccc} L_{B_{\mathrm{noise}}^{1}} & = & \sum_{i=1}^{3}\gamma_{i}\sigma_{i}\rho \sigma_{i}^{†}-\sum_{i=1}^{3}\gamma_{i}\rho \\ & = & \gamma_{\mathrm{relax}}\sum_{s=\pm }\sigma_{s}\rho \sigma_{s}^{†}+\gamma_{\mathrm{deph}}\sigma_{3}\rho \sigma_{3}^{†}-\left(\gamma_{\mathrm{relax}}+\gamma_{\mathrm{deph}}\right)\rho \end{array}$$

with $\gamma_{\mathrm{deph}}=\gamma_{3}$, the corresponding coefficients for relaxation noise and dephasing noise. They are responsible for the energy relaxation (loss or gain) and pure dephasing (decoherence without energy change), respectively. The property Equation (24) becomes extended for processes involving more qubits. The PL noise model then equalises the loss or damping ($\sigma_{-}$) and the gain ($\sigma_{+}$) of energy. In fact, considering Equation (25), it is easy to demonstrate that $\frac{\partial \bar{E}}{\partial t}=\frac{\partial \mathrm{Tr}(H\rho )}{\partial t}=\left(E_{1}-E_{0}\right)\gamma_{\mathrm{relax}}\left(\rho_{00}-\rho_{11}\right)$, which has an asymptotic solution for t→∞: $\rho_{00},\rho_{11}\to \frac{1}{2},\bar{E}\to \frac{1}{2}\left(E_{0}+E_{1}\right)$. We have developed our analysis on the basis of |0〉and|1〉; however, for PL noise models with equal coefficients $\gamma_{i}=\gamma$, it becomes invariant under an orthonormal basis change, implying that our previous analysis fits for them with $\gamma_{\mathrm{relax}}=2\gamma_{\mathrm{deph}}$.

For two qubits, if $\gamma_{i,1}=\gamma_{i,2}$ and $\gamma_{1,i}=\gamma_{2,i}$, a similar but more complex property is fulfilled, but in this case we can also have relaxation in one qubit together with dephasing in another, at least if $\gamma_{ij}=0$ for all i∈{1,2},j=3 or j∈{1,2},i=3. Again, pure dephasing noise does not contribute to the energy change. In addition, if the coefficients $\gamma_{ij}$ become equal (as in this work), the PL noise model also becomes invariant under an orthonormal change of basis (see Appendix A).

## 4. Thermalisation of Universal Quantum Gates

The thermalisation of the universal quantum gates process describes how gate operations, interacting with an environment, transition into mixed or Gibbs-like steady states instead of maintaining ideal unitary states. Within the LME framework, this behaviour can be formalised: under quantum detailed balance conditions, the non-equilibrium steady state acquires a Gibbsian form with an effective Hamiltonian [54].

This section discusses thermalisation of magnetic or electric dipoles by a thermal bath, characterised by its emission of blackbody radiation. Such an interaction leads to absorption and emission processes governed by the Bose-Einstein photon distribution [43], driving the system away from ideal unitaries and towards Gibbs-like steady states [55,56]. Blackbody-induced transitions in the atomic and Rydberg systems reveal how thermal photons drive population transfer, dephasing, and state mixing, offering evidence of bath-driven thermalisation [57,58].

### 4.1. Thermalisation on Single-Qubits Being Processed by One-Qubit Gate

We will consider another type of decoherence produced by thermalisation. Consider the Hamiltonian Equation (2), which ideally reaches the set of universal gates $U_{\pi /4},U_{\pi /8},H$ whose prescriptions were given in Equations (9)–(11). Although each gate is achieved in a characteristic time $T_{G}$, we have already rescaled the time as $\tau =\frac{t}{T_{G}}$. However, the dissipation times $T_{P}$ when the system reaches the thermal equilibrium Gibbs state $\rho_{G}=e^{\beta H}/\mathrm{Tr}\left(e^{\beta H}\right)$ ($\beta^{-1}=k_{B}T$, being the Boltzmann constant) are often much longer than $T_{G}$. In this analysis, we follow the development reached in [59] for an electric or magnetic dipole within a blackbody radiation thermal bath at temperature *T*. The previous development assumes a time-independent Hamiltonian. In the current development, it is applicable because $T_{G}\ll T_{P}$ is assumed. Then, we can approximate the thermalisation process considering the $\bar{\Omega }$ average as Ω constant because of its continuity. In this approximation, in terms of [59], considering that the normalised time is τ, the eigenvalues are $\epsilon_{i}=\pm \frac{1}{2}\hbar \bar{\Omega }=\pm \frac{1}{2}\hbar \theta \left(1\right)$.

Thus, depending on the gate in Equations (9)–(11), we need to choose the direction of interaction $\hat{n}$, which implies that the energy eigenstates will be $|\epsilon_{i}\rangle ,i=0,1$ for each of the following: (a) |±〉 for $\hat{ı}$, (b) $|Y_{\pm }\rangle$ for $\hat{j}$, (c) |i〉,i=0,1 for $\hat{k}$, and (d) $|h_{\pm }\rangle \equiv \frac{1\mp \sqrt{2}}{\sqrt{2(2\mp \sqrt{2})}}|0\rangle +\frac{1}{\sqrt{2(2\mp \sqrt{2})}}|1\rangle$, the eigenvectors of the Hadamard operator. In any case, there are two Lindblad operators, $L_{01}=l_{01}|\epsilon_{0}\rangle \langle \epsilon_{1}|$ and $L_{10}=l_{10}|\epsilon_{1}\rangle \langle \epsilon_{0}|$, which require the construction of a series of values $D_{ij},C_{ij}$. Following [59], the corresponding values $D_{01},D_{10}$ become independent of the $\hat{n}$ election:

(26) $$\begin{array}{ccc} D_{ij} & = & g\sum_{k=1}^{3}|\langle \epsilon_{i}|\sigma_{k}|\epsilon_{j}{\rangle |}^{2}=2g\\ & & \mathrm{with}:\;g=\frac{4K_{i}\mu^{2}}{3\hbar^{4}c^{3}} \end{array}$$

$K_{i}=K_{e}=\frac{1}{4\pi \epsilon_{0}}$ if the dipole is electric, or $K_{i}=K_{m}=\frac{\mu_{0}}{4\pi }$ for a magnetic dipole. In this case, we are using SI units. Thus, the square amplitudes of $l_{ij}$ become the following [59]:

(27) $$\begin{array}{ccc} |l_{ij}{|}^{2} & = & \frac{|\epsilon_{i}-\epsilon_{j}{|}^{3}e^{-\frac{\beta (\epsilon_{i}-\epsilon_{j})}{2}}}{2\sinh\frac{\beta |\epsilon_{i}-\epsilon_{j}|}{2}}=g\hbar^{3}{\left|\bar{\Omega }\right|}^{3}e^{{\left(-1\right)}^{i}\frac{\beta \hbar |\bar{\Omega }|}{2}}\mathrm{csch}\frac{\beta \hbar |\bar{\Omega }|}{2} \end{array}$$

while the dissipation and decoherence times, $T_{P}$ and $T_{Q}$, respectively, are obtained similarly [59]:

(28) $$\begin{array}{ccc} T_{P} & = & \frac{1}{2}T_{Q}=\frac{\tanh\frac{\beta \hbar |\bar{\Omega }|}{2}}{2g\hbar^{3}{\left|\bar{\Omega }\right|}^{3}}=\frac{\tanh E}{\Theta^{3}} \end{array}$$

Introducing the two parameters $E=\frac{\beta \hbar |\bar{\Omega }|}{2}$ and $\Theta ={\left(2g\right)}^{\frac{1}{3}}\hbar \left|\bar{\Omega }\right|$ (still, it should be considered that Θ≪1 to fulfil $T_{G}\ll T_{P}$), these last expressions become (which could be considered real without loss of generality):

(29) $$\begin{array}{ccc} l_{01}^{2} & = & \frac{1}{T_{P}}\frac{e^{2E}}{1+e^{2E}} \end{array}$$

(30) $$\begin{array}{ccc} l_{10}^{2} & = & \frac{1}{T_{P}}\frac{1}{1+e^{2E}} \end{array}$$

Noting that E represents the relative energy between the gap energy of the eigenvalues and the thermal energy $k_{B}T$. It is inversely proportional to *T*. In addition, due to Equation (29), $T_{P}$ could be expressed on the same scale as τ, thus implying that $1\ll T_{P}=\frac{1}{2}T_{Q}$. This procedure provides a complete description of the Lindblad coefficients, without requiring the coefficient γ (noticing that $l_{ij}$ already includes the correct physical dimensions needed in the Lindblad equation as a function of temperature). A factor $\Theta^{3}$ can be obtained in Equation (15), $\Gamma =\Theta^{3}$. Note that a longer dissipation time reduces the thermal decoherence effect due to a faster gate.

**Figure 7:**
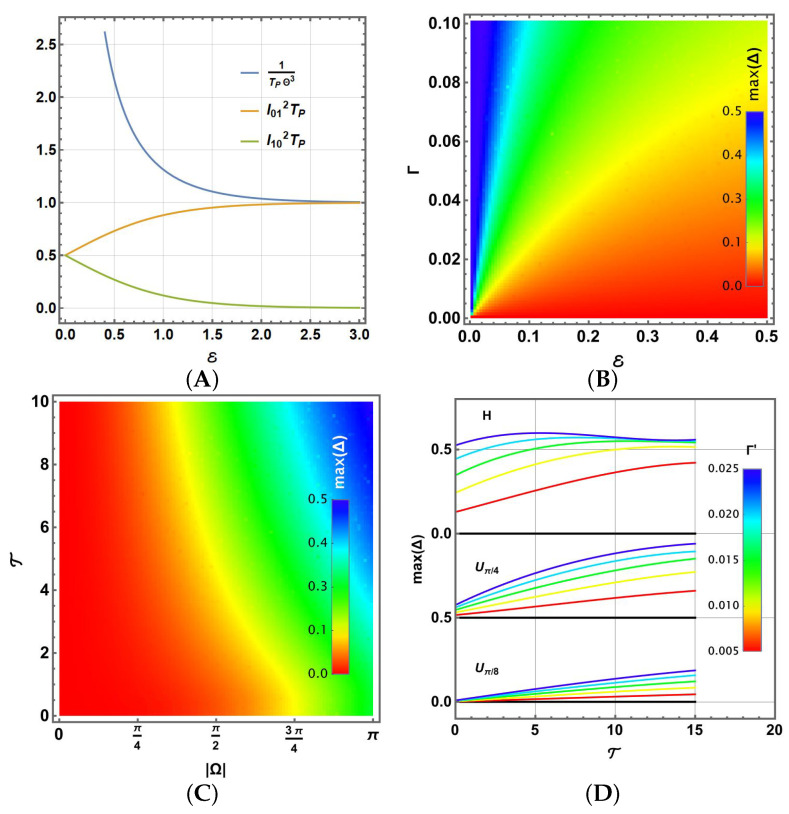
(**A**) Behaviour of $l_{01}^{2}T_{P},l_{10}^{2}T_{P}$ and $\frac{1}{T_{P}\Theta^{3}}$ as a function of E. max(Δ) on the Bloch sphere as a function of (**B**) E and Γ for $U_{\pi /8}$, (**C**) of $|\bar{\Omega }|$ and T for $\Gamma^{\prime }=0.01$, and (**D**) of $\Gamma^{\prime }$ and T for each universal one-qubit gate.

However, if *T* increases, then E→0 and $T_{P}\to 0$, exhibiting a greater impact on the dissipation process. These quantities are represented in Figure 7A, showing the individual contributions of $l_{01}^{2}T_{P}$ and $l_{10}^{2}T_{P}$, and the common factor $\frac{1}{T_{P}}$ (normalised by $\Theta^{3}$). Thus, if E→0 (T→∞), then $l_{ij}^{2}T_{P}\to \frac{1}{2}$, showing the equitable contribution of the operators |i〉〈j|,i≠j required to reach the Gibbs distribution for higher temperatures. Instead, if E→∞ (T→0), $l_{10}^{2}\to 0$ and $l_{01}^{2}\to 1$, the transition to the ground state is privileged, as the Gibbs state requires for T→0. This representation allows us to notice that $G_{\mathrm{thermal}}^{1}=\frac{1}{T_{P}}=\Theta^{3}\coth E=2g\hbar^{3}{\left|\bar{\Omega }\right|}^{3}\coth\frac{\beta \hbar |\Omega |}{2}$ becomes comparable to the previous $3\gamma ,\gamma_{\max},9\Gamma$ general coefficients.

Another useful analysis results from parameterising previous expressions using $|\bar{\Omega }|$, $T=\frac{k_{B}T}{\hbar }$, and the constant $\Gamma^{\prime }=2g\hbar^{3}$. They become the following:

(31) $$\begin{array}{ccc} l_{01}^{2} & = & \Gamma^{\prime }{\left|\bar{\Omega }\right|}^{3}\frac{e^{|\bar{\Omega }|/T}}{e^{|\bar{\Omega }|/T}-1} \end{array}$$

(32) $$\begin{array}{ccc} l_{10}^{2} & = & \Gamma^{\prime }{\left|\bar{\Omega }\right|}^{3}\frac{1}{e^{|\bar{\Omega }|/T}-1} \end{array}$$

This parameterisation allows us to observe separately the contribution of $|\bar{\Omega }\left|=|\theta \left(1\right)\right|$ and the rescaled temperature T. Thus, for comparison, we report in Figure 7B,C the highest values of Δ on the Bloch sphere states (obtained with the Monte Carlo method) due to the thermalisation of single gates for both sets of parameters. Figure 7B shows the results for the $U_{\pi /8}\left(\right|\bar{\Omega }\left|=\theta \left(1\right)=\frac{\pi }{4}\right)$ gate. The results for the $U_{\pi /4}$ and Hadamard gates are similar, with both Γ and E two- or four-fold, respectively, thus maintaining the structure and aspect of the colour plot. It shows how increasing E reduces errors, but they are still increased by Γ. In this case, the comparison should be understood as fixing $|\bar{\Omega }|$. In this sense, the relative energy maintains the precision on the gate, not merely the reduction of *T*, although Γ still depends on the amplitude $|\bar{\Omega }{|}^{3}$.

By analysing the behaviour in terms of $|\bar{\Omega }|$ versus T in Figure 7C, we obtain the analysis for all $U_{\theta (1)/2}$ single-qubit gates, in particular, those for $\theta \left(1\right)=\frac{\pi }{4}\left(U_{\pi /8}\right)$, $\theta \left(1\right)=\frac{\pi }{2}\left(U_{\pi /4}\right)$ and θ(1)=π (Hadamard) in agreement with the prescriptions (9)–(11). It shows for overall values of $|\bar{\Omega }\left|=|\theta \left(1\right)\right|$ that only the lowest values of T allow low errors, but still lower values of $|\bar{\Omega }\left|=|\theta \left(1\right)\right|$ with increasing temperatures due to the factor $|\bar{\Omega }{|}^{3}$. Here, we have used $\Gamma^{\prime }=0.01<\frac{1}{\pi^{3}}$ to facilitate the comparison with Figure 7B, but note that its increase/decrease leads to higher/lower results for max(Δ). Finally, Figure 7D shows the full dependence for $\Gamma^{\prime }$ (shown in colour in agreement with the legend) and T for each gate, noticing that, as expected, max(Δ) increases with the increase in both parameters. There, it is also observed that a higher value of $|\bar{\Omega }|$ produces a higher negative effect on the gate due to thermalisation. Moreover, note that higher values of $\Gamma^{\prime }$ for the Hadamard gate produce higher values than max(Δ)>0.5, which goes asymptotically to max(Δ)=0.5 only if T→∞. Instead, note that when $\Gamma^{\prime }$ increases, clearly max(Δ)≠0 when T→0, due to radiative effects.

### 4.2. Thermalisation of CNOT Gate

In this section, we use the previous procedure for thermalisation for two distinguishable qubits [59], following a procedure similar to that in Section 3. In this case, the Cartan decomposition is used in terms of the Hamiltonian Equation (21). As before, assuming $T_{G}\ll T_{P}$, each step in the Hamiltonian is considered as an average field $\bar{\Omega }\left(t\right),J$:

(33) $$\begin{array}{c} H\left(t\right)=\left\{\begin{array}{cc} +\frac{\hbar \pi }{4}\sigma_{0}^{1}⊗\sigma_{2}^{2} & ,\;\tau \in [0,1)\\ -\frac{\pi \hbar }{4}\sigma_{3}^{1}⊗\sigma_{3}^{2} & ,\;\tau \in [1,2)\\ -\frac{\hbar \pi }{4}\sigma_{0}^{1}⊗\sigma_{3}^{2} & ,\;\tau \in [2,3)\\ -\frac{\hbar \pi }{4}\sigma_{0}^{1}⊗\sigma_{2}^{2} & ,\;\tau \in [3,4)\\ +\frac{\hbar \pi }{4}\sigma_{3}^{1}⊗\sigma_{0}^{2} & ,\;\tau \in [4,5] \end{array}\right. \end{array}$$

In any step of this approximation, the energy is two-fold degenerate ($E_{1}=E_{2}<E_{3}=E_{4}$), reducing the number of non-zero Lindblad jump operators $L_{ij}=l_{ij}|\epsilon_{i}\rangle \langle \epsilon_{j}|,\;i,j=1,\ldots ,4$, with $l_{ij}\neq 0$ for $E_{i}\neq E_{j}$. Moreover, because all Hamiltonian strengths are $\pm \frac{\hbar \pi }{4}$, a direct calculation shows (by first calculating $D_{ij},C_{ij}$ as in [59]) that there are only two possible values for $l_{ij}^{2}=\frac{1}{8}g\pi^{3}\hbar^{3}e^{-\mathrm{sgn}\left(E_{i}-E_{j}\right)\frac{\hbar \pi }{4}}\mathrm{csch}\frac{\hbar \pi }{4}$ (sgn is the sign function). With these results, we can numerically solve the Lindblad equation in t→τ∈[0,5] as in Equation (3), and we can reach analytical values for $T_{Q},T_{P}$ in each time step. In fact, again considering the variable $T=\frac{k_{B}T}{\hbar }$ and the constant $\Gamma^{\prime \prime }=g\hbar^{3}$, then we obtain the following:

(34) $$\begin{array}{ccc} & & 1\ll T_{Q_{\mathrm{loc}}}=T_{P_{\mathrm{loc}}}=\frac{4\tanh\frac{\pi }{4T}}{g\hbar^{3}\pi^{3}} \end{array}$$

(35) $$\begin{array}{ccc} & & 1\ll T_{Q_{\mathrm{non}-\mathrm{loc}}}=\frac{4\tanh\frac{\pi }{4T}}{g\hbar^{3}\pi^{3}}<\frac{4}{g\hbar^{3}\pi^{3}\left(\coth\frac{\pi }{4T}-1\right)}=T_{P_{\mathrm{non}-\mathrm{loc}}} \end{array}$$

where loc refers to local single-qubit rotations and non-loc to Ising-like interactions. Note that only $T_{P_{\mathrm{loc}}}$ takes the same value as $T_{P}$ for the single-qubit gate involved, $U_{\pi /4}$, but not $T_{Q_{\mathrm{loc}}}<T_{Q}$. In the current analysis, we have several reduced critical parameters, $\Gamma^{\prime \prime },T$, together with the entire distribution of two-qubit states, represented by the states with real coefficients given by Equation (22). Unlike the noise analysis for the CNOT gate, where the parameter Γ plays the global role of decoherence, in this case $\Gamma^{\prime \prime }$ takes a partial role for decoherence, while T in $l_{ij}$ provides a balance between the jumps between the allowed states for the two energies involved.

Moreover, a brief analysis shows that each step in Cartan decomposition has a different number of allowed transitions ($l_{ij}^{2}\neq 0$), four for the single-qubit rotations and eight for the Ising-like interaction. Two different global Lindblad coefficients are obtained, $G_{\mathrm{thermal}}^{2,1}=4\Gamma^{\prime \prime }{\left(\frac{\pi }{2}\right)}^{3}\coth\frac{\beta \hbar \pi }{4}=4g\hbar^{3}{\left(\frac{\pi }{2}\right)}^{3}\coth\frac{\beta \hbar \pi }{4}=4g\hbar^{3}{\left|\bar{\Omega }\right|}^{3}\coth\frac{\beta \hbar |\bar{\Omega }|}{2}$ for rotations and $G_{\mathrm{thermal}}^{2,2}=8\Gamma^{\prime \prime }{\left(\frac{\pi }{2}\right)}^{3}\coth\frac{\beta \hbar \pi }{4}=8g\hbar^{3}{\left(\frac{\pi }{2}\right)}^{3}\coth\frac{\beta \hbar \pi }{4}=64g\hbar^{3}J^{3}\coth\beta \hbar J$ for the Ising-like interaction. They are comparable to the previous $3\gamma ,\gamma_{\max},9\Gamma$ and $\Gamma^{\prime }{\left|\Omega \right|}^{3}\cot\frac{\beta \hbar |\bar{\Omega }|}{2}$ obtained in the previous analyses. These selections let the remaining individual coefficients in each $l_{ij}^{2}$ add one as in the previous cases (as in Figure 7A).

**Figure 8:**
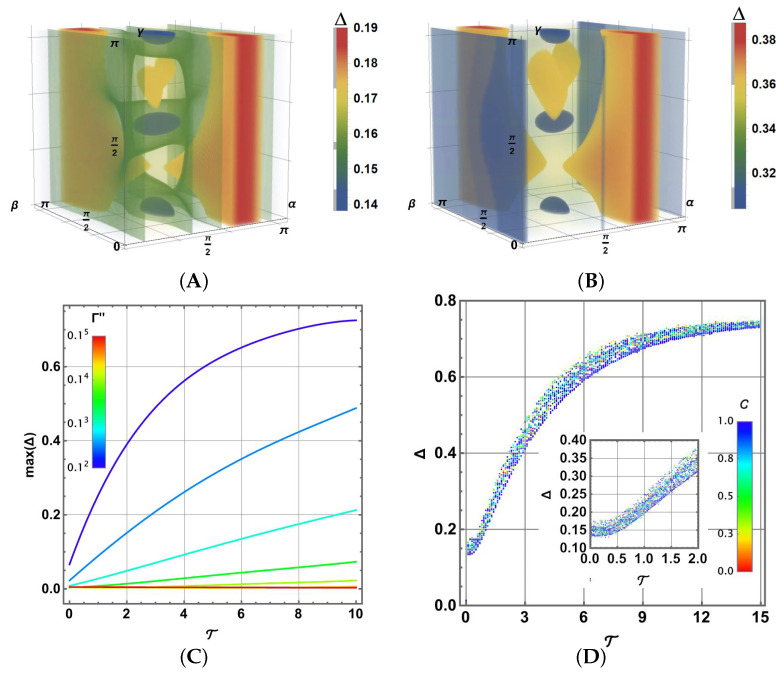
Δ distribution on initial states $\rho_{0}$ in the α,β,γ parametric space for (**A**) $\Gamma^{\prime \prime }=0.01,T=0.5$ and (**B**) $\Gamma^{\prime \prime }=0.01,T=2.0$. (**C**) max(Δ) on the parametric space of initial states as a function of T, for several values of $\Gamma^{\prime \prime }=0.1^{2.0},0.1^{2.5},\ldots ,0.1^{5.0}$. (**D**) Dispersion of Δ values for $\Gamma^{\prime \prime }=0.001$ between T∈[0,15] coloured in agreement with the concurrence bar scale together.

Figure 8A,B illustrate the distributions of Δ for $\Gamma^{\prime \prime }=0.01$ with T=0.5 and T=2.0, respectively, as a density plot in the parametric space of the states in Equation (22). Each dot in the parametric space has been coloured in agreement with Δ as the legend bar sets. As before, density plots show in solid colours the Δ values marked with a vertical grey bar to the left of the scale. Other colours are transparently suggested. For the lower temperature T in Figure 8A, there is a rich structure that changes slightly to that in Figure 8B. Compared to the guide map in Figure 6A, there is a less clear correlation with initial entanglement (as was the case for the noisy case, Δ becomes larger for separable states). Note that the range of the Δ scale slightly decreases with increasing temperature. It is noticeable that the lower errors due to thermalisation (blue) occur for the states nearest to the Bell states.

In a more synthetic but wider view, Figure 8C shows $\max\left(\Delta \right)=\max_{\begin{array}{c} \left(\alpha ,\beta ,\gamma \right)\in {\left[0,\pi \right]}^{\times 3} \end{array}}\left\{\Delta \right\}$ (obtained using the Monte Carlo method) as a function of T for several values of $\Gamma^{\prime \prime }=0.1^{2},\ldots ,0.1^{5}$ (shown in colour, in agreement with the included scale). In this case, max(Δ) becomes asymptotic to 0.75 as a function of T. Both cases shown in Figure 8A,B are contained in the blue curve for Γ=0.01 at T=0.5,2.0. Note how the increase in T increases in max(Δ), but also higher values of $\Gamma^{\prime \prime }$ worsen the imperfect results of the CNOT gate. In fact, the thermalisation impact of $\Gamma^{\prime \prime }$ becomes larger than that of $\Gamma^{\prime }$ for the one-qubit gates; note in particular that an exponential scale has been needed to effectively show the variability in $\Gamma^{\prime \prime }$. Note that for T→0, max(G)≠0 because of the radiative effects.

Alternatively, Figure 8D shows the case $\Gamma^{\prime \prime }=0.01$ for a sample of $10^{4}$ random values in the interval T∈[0,15] and throughout the state space ${\left[0,\pi \right]}^{\times 3}$. Each outcome has been coloured in agreement with its initial value of concurrence. As noted in Figure 8A,B, there is no clear correlation between initial entanglement and the final gate error, although in this case the separable states (C=1) have shown a different occupation of the largest values Δ. Of special interest is the radiative behaviour observed in the inset for T≈0 for the initial growth in Δ. This behaviour is also observed for other values of $\Gamma^{\prime \prime }$ in Figure 8C, but they also occupy short intervals near T=0 and are almost not noticed because of the slower growth of Δ when T increases. Finally, as in the previous analysis for noise and thermalisation, the range of Δ dispersion is reduced when T→∞ due to asymptotic behaviour and achievement of the Gibbs state.

## 5. Discussion

In the previous sections, we have analysed under a general approach the modelling of noise and thermalisation of quantum universal gates using a Lindbladian approach. The meaningful contribution to these noisy effects does not depend on the input state processed but instead on the overall coefficients involved in the Lindblad operators, each having been characterised by global and individual transition coefficients. The global coefficients introduced through development, $G_{\mathrm{noise}}^{1},G_{\mathrm{noise}}^{2},G_{\mathrm{thermal}}^{1},G_{\mathrm{thermal}}^{2,1},G_{\mathrm{thermal}}^{2,2}$, when increasing, naturally generate a greater alteration in the gate performance almost independently of the input state. For the thermalisation cases, the general coefficients increase as a function of temperature, a fact that is naturally expected because cothβE→∞ if T→0. In addition, in those cases, the individual remaining coefficients in each transition $|\epsilon_{i}\rangle \langle \epsilon_{j}|$ provide a differentiated weight as a function of temperature (which adds one).

Note that for noise, $G_{\mathrm{noise}}^{1}\equiv 3\gamma ,G_{\mathrm{noise}}^{2}=\Gamma$ were modelled identically without a physical basis. Instead, for $G_{\mathrm{thermal}}^{1}=2g\hbar^{3}{\left|\bar{\Omega }\right|}^{3}\coth\frac{\beta \hbar |\bar{\Omega }|}{2}$ with $|\bar{\Omega }|=\frac{\pi }{4},\frac{\pi }{2},\pi$ as a function of the gate, we notice that these values consecutively scale by a factor greater than eight (because of the increase in $\coth\frac{\beta \hbar |\bar{\Omega }|}{2}$). In addition, $G_{\mathrm{thermal}}^{2,1}=4g\hbar^{3}{\left|\bar{\Omega }\right|}^{3}\coth\frac{\beta \hbar |\bar{\Omega }|}{2}<G_{\mathrm{thermal}}^{2,2}=64g\hbar^{3}J^{3}\coth\beta \hbar J$ for $|\bar{\Omega }|=2J=\frac{\pi }{2}$. Hence, $G_{\mathrm{thermal}}^{1}=\frac{1}{2}G_{\mathrm{thermal}}^{2,1}=\frac{1}{4}G_{\mathrm{thermal}}^{2,2}$ in this case. They depend on several physical parameters, $T,|\bar{\Omega }|$ or J, and dipole momentum μ (through *g*). In summary, the report of $\Delta^{1}\equiv \max\left(\Delta \right)$ for $G_{\mathrm{thermal}}^{1}$ with $|\bar{\Omega }|=\frac{\pi }{2}$ and $\Delta^{2}\equiv \max\left(\Delta \right)$ for $G_{\mathrm{thermal}}^{2,2}$ with $J=\frac{\pi }{4}$, respectively, for one or two qubit gates, represents characteristic values of Δ for particular related technologies.

Although in current applications for quantum computation, technologies use several interactions, not necessarily magnetic or electric dipoles; however, several technologies are truly based on them. There, T≈10–20 mK on average (using a dilution refrigerator, a cryostat using a helium dilution process to cool quantum processors to near absolute zero), but there are still other neutral atom technologies holding atoms in optical traps and laser cooling, reaching temperatures of order μK [60]. There, the associated Lindblad coefficients $G_{\mathrm{real}}$ are obtained experimentally by measuring relaxation times [45,61]. For them, we can compare their general behaviour by translating their reported parameters into the literature to set an estimation for Δ based on our procedures. Thus, considering $G\equiv g\hbar^{3}{\left|\bar{\Omega }\right|}^{3}\coth\frac{\beta \hbar |\bar{\Omega }|}{2}$ as a characteristic value G associated with one or two qubit gates, we will estimate for several technologies some related parameters included in our procedures, together with the corresponding values $\Delta^{1}$ and $\Delta^{2}$ for $G_{\mathrm{thermal}}^{1}$ and $G_{\mathrm{thermal}}^{2,2}$, respectively, as previously defined (in fact, max(Δ) will be reported on the Bloch sphere).

Table 1 reports the physical energy transition frequency |Ω|=ω (not scaled, see Appendix B), the operating temperature *T*, and the physical $G_{\mathrm{real}}$ experimentally estimated from the decay rates for some of these technologies (only magnitudes are commonly reported, comprising several decoherence processes, not only thermal). Dimensionless E is easily calculated from these data (clearly independent of scaling), as well as scaled T and $G_{\mathrm{sc}}$, then $G_{\mathrm{nsc}}$ (non-scaled). Scaling is performed in all time units, in any case, with the factor $T_{G}=\frac{\pi }{2\Omega }$ (gate operation time). For comparison, we have chosen $U_{\pi /4}$ because it has $|\bar{\Omega }|=\frac{\pi }{2}$, comparable to the parameter J in Ising-like interactions and the rotations implemented in Cartan decomposition for the CNOT gate. This allows us to easily relate $G_{\mathrm{thermal}}^{2,1}$ with $G_{\mathrm{thermal}}^{2,2}$ for the case of two qubits (as previously established).

Thus, we can numerically estimate $\Delta^{1}$ and $\Delta^{2}$ (respectively, corresponding to our one-qubit and two-qubit gate models through $G_{\mathrm{thermal}}^{1}=2G$ and $G_{\mathrm{thermal}}^{2,1}=8G$) corresponding to the parameters in our development for a similar parameter dipole-based quantum system. Note that $G_{\mathrm{real}}$ is included only for comparison with $G_{\mathrm{nsc}}$. It is noticeable that three technologies have really low $G_{\mathrm{sc}}$ values, maintaining thermalisation in the region with minimal impact on max(Δ) (for example, see Figure 7B and Figure 8C), inclusively for two-qubit gates. In particular, note that, at least for the first two technologies, Rydberg and QED, cothE≈1, then scaled $\Gamma ,\Gamma^{\prime \prime }$ remain in the same order of magnitude as $G_{\mathrm{sc}}$. However, they become only an approximation because not all potential sources of noise are considered.

**Figure 9:**
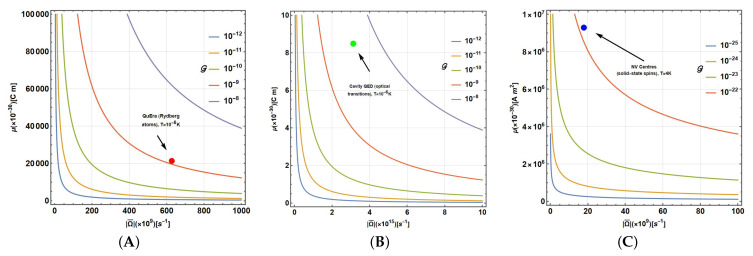
Contour plots of G as function of $|\bar{\Omega }|$ and μ showing the position of some technologies: (**A**) QuEra (Rydberg atoms), at $T\approx 10^{-6}$ K (electric dipoles), (**B**) Cavity QED (optical transitions), at $T\approx 10^{-6}$ K (electric dipoles), and (**C**) NV Centres (solid-state spins), at T≈4 K (magnetic dipoles).

Hence, as a final analysis, we can locate the technologies listed in Table 1—Rydberg atoms, Nitrogen Vacancy (NV) centres in diamonds, and optical transitions in cavity Quantum Electrodynamics (QED)—based on dipolar interactions (NV centres and trapped ions are magnetic; others are electric) and their relative position in a map for $G_{\mathrm{nsc}}$. Using the energy transition reported for each system $\hbar |\bar{\Omega }|$ together with the respective dipolar momenta μ (electric or magnetic) obtained directly from physical constants (electron charge, Bohr radius, Bohr magneton, etc., see Appendix B), $G_{\mathrm{nsc}}=g\hbar^{3}{\left|\bar{\Omega }\right|}^{3}\coth\frac{\beta \hbar |\bar{\Omega }|}{2}$ is schematically shown in Figure 9. It appears as a function of $|\bar{\Omega }|$ (in $s^{-1}$, on a scale of $10^{9}$) and μ (on a scale of $10^{-30}$ using the corresponding units for electric or magnetic dipoles.

For Rydberg atoms in QuEra technologies [60] ($10^{-6}$K, $\hbar |\bar{\Omega }|=6.28\times 10^{11}s^{-1}$), $G_{\mathrm{sc}}\approx 10^{-9}$–$10^{-8}$ (Figure 9A) as shown by the red dot on the graph. Figure 9B corresponds to Figure 9A ($10^{-6}$K, $\hbar |\bar{\Omega }|=3.14\times 10^{15}$$s^{-1}$) on a different scale to show the QED technologies in cavities [62] with $G_{\mathrm{sc}}\approx 10^{-9}$–$10^{-8}$ (green dot). Figure 9C shows the plot for the NV centres [63] (in 4 K, $\hbar |\bar{\Omega }|=18\times 10^{9}$ $s^{-1}$) with $G\approx 10^{-22}$–$10^{-21}$c (blue dot), showing very little thermal decoherence due to the weak magnetic interaction (observe the comparison between $G_{\mathrm{real}}$ and $G_{\mathrm{nsc}}$, which shows the role that a radiation field plays for thermalisation in each type of system). Nevertheless, NV centres are based on condensed matter, so the approximation for the thermal bath only as a radiation field is extremely mild. In general, the values obtained are extremely small; nevertheless, other decoherence effects are commonly concomitant (scattering, parasitic radiation, Purcell effect, etc.), thus raising them (particularly phononic baths for NV centre technologies). Finally, using the Fuchs-van de Graaf inequality, the fidelity ranges for single-qubit gates for each technology (Rydberg atoms, NV centres, and QED optical transitions) become 0.998–0.999 (using $\Delta^{1}$ from Table 1) for three technologies. For two-qubit gates, for the same technologies, fidelities are in the ranges 0.970–0.999 (using $\Delta^{2}$ from Table 1).

## 6. Conclusions

We have presented a Lindblad-based model to analyse some effects generating decoherence as digital noise and thermalisation on qubits being controlled by individual elements selected among a set of universal quantum gates. We observed that the effects of damping and dephasing arise when modelling noise and thermalisation. We have analysed how different magnitudes of decay rates $\gamma_{i}$ affect the deviation of quantum states from the ideal unitary evolution expected by each controlled quantum gate, quantified by the trace distance. The trace distance estimation can be translated to a fidelity range with the Fuchs-van de Graaf inequality to assess this noise indicator commonly employed in this topic. Our gate deviation simulations show a certain dependence on initial entanglement but a clear dependence on the magnitude of the decay rates, even though they are almost indistinct from the specific states being processed. This work has only considered the effects of noise and thermalisation on decoherence; however, other factors such as the Purcell effect [64,65], relaxation from intrinsic losses [66,67,68] or pure dephasing [69] add to the global effect.

As seen, the Lindbladian framework provides a consistent and flexible approach to capture the essential features of open quantum systems and their behaviour in noisy or thermal environments. By applying it across a diverse range of parameter values, we established a systematic way to assess the impact of noise channels on the performance of quantum circuits. Thermalisation models provide a detailed physical reference for the temperature effects on the quantum gates’ functioning, including radiative ones. This methodology offers a useful tool for theoretical benchmarking on error algorithms, independent of specific hardware platforms.

Future work could extend this analysis by incorporating correlated noise channels or device-specific error channels. In particular, benchmarking with real cloud back-ends could provide a direct comparison between simulated Lindblad models and observed hardware performance. Such studies would support the continuous development of error mitigation strategies, particularly those that move general noise to Pauli–Lindblad noise, for example, by Pauli twirling, to then apply PEC, ZNE, TEM, or other similar QEM techniques. Inclusion and addition of complementary sources of decoherence are also mandatory. In addition, other specific Hamiltonians for alternative quantum computation models should be considered, together with the analysis of post-quantum parallel strategies for minimising these undesirable effects by exploiting the same quantum nature of the systems, particularly by noticing the low impact that concrete initial states have in the process.

## Figures and Tables

**Table 1:** Some operation parameters of selected quantum computer technologies and $\Delta^{1},\Delta^{2}$ are numerically calculated for a comparable dipole-based system through formulas for $G_{\mathrm{thermal}}^{1}$ and $G_{\mathrm{thermal}}^{2,2}$.

Technology	$\left|\Omega |(s^{-1}\right)$	T(K)	$G_{\mathrm{real}}\;$($s^{-1}$)	E	T	$G_{\mathrm{sc}}$	$G_{\mathrm{nsc}}\;$($s^{-1}$)	$\Delta^{1}$	$\Delta^{2}$
QuEra (Rydberg atoms) [60]	$6.3\times 10^{11}$	$1.0\times 10^{-6}$	$\approx 10^{4}$	$2.4\times 10^{6}$	$3.3\times 10^{-7}$	$1.2\times 10^{-9}$	$4.7\times 10^{2}$	$7.92\times 10^{-4}$	$1.53\times 10^{-2}$
Cavity QED (optical transitions) [62]	$3.1\times 10^{15}$	$1.0\times 10^{-6}$	$\approx 10^{6}$	$1.2\times 10^{10}$	$6.5\times 10^{-11}$	$4.7\times 10^{-9}$	$9.4\times 10^{6}$	$7.93\times 10^{-4}$	$1.55\times 10^{-2}$
NV Centres (solid-state spins) [63]	$1.8\times 10^{10}$	$4.0\times 10^{0}$	$\approx 10^{4}$	$1.7\times 10^{-2}$	$4.6\times 10^{1}$	$1.2\times 10^{-22}$	$1.4\times 10^{-12}$	$7.90\times 10^{-4}$	$1.50\times 10^{-2}$

## Data Availability

No new data were created or analyzed in this study. Data sharing is not applicable to this article.

## Abbreviations

The following abbreviations are used in this manuscript:

LME	Lindblad Master Equation
NISQ	Noisy Intermediate-Scale Quantum
NV	Nitrogen Vacancy
PEC	Probabilistic Error Cancellation
PL	Pauli–Lindblad Noise Model
QED	Quantum Electrodynamics
QEM	Quantum Error Mitigation
SI	Units International System
TEM	Tensor-Network Error Mitigation
WURST	Wideband, Uniform Rate, Smooth Truncation
ZNE	Zero Noise Extrapolation

## Appendix A. Invariance of PL Noise Model

Consider two different representations of the Pauli operators and identity, $\left\{\sigma_{\alpha }|\alpha =0,\ldots ,3\right\}$ and $\left\{\Sigma_{\alpha }|\alpha =0,\ldots ,3\right\}$, both related by an orthonormal combination: $\sigma_{\alpha }=\sum_{\beta =0}^{3}s_{\alpha \beta }\Sigma_{\beta }$. Here, $\sigma_{0}=\Sigma_{0}$ represents the identity. It implies that: (a) $s_{00}=1$, (b) $s_{0i}=1,i=1,\ldots ,3$. In the following, Latin subscripts take only 1,…,3 values (Pauli operators), and Greek subscripts take the overall 0,…,3 values. Thus, $\sigma_{i}=\sum_{j=1}^{3}s_{ij}\Sigma_{j}$. Noticing that:

(A1) $$\begin{array}{c} 3\sigma_{0}=\sum_{i=1}^{3}\sigma_{i}^{2}=\sum_{j,k=1}^{3}\left(\sum_{i=1}^{3}s_{ij}s_{ik}^{*}\right)\Sigma_{j}\Sigma_{k}^{†}\to \sum_{i=1}^{3}s_{ij}s_{ik}^{*}=\delta_{jk} \end{array}$$

because the transformation is orthonormal. Then, it implies that:

(A2) $$\begin{array}{c} \sum_{i=1}^{3}\sigma_{i}\rho \sigma_{i}^{†}=\sum_{i=1}^{3}\sum_{j,k=1}^{3}s_{ij}s_{ik}^{*}\Sigma_{j}\rho \Sigma_{k}^{†}=\sum_{k=1}^{3}\Sigma_{k}\rho \Sigma_{k}^{†} \end{array}$$

which implies that the PL noise model with equal coefficients $\gamma_{i}=\gamma$ becomes invariant under a basis change.

## Appendix B. Electric and Magnetic Dipole Moments Calculations for Some Current Technologies

This section includes calculations of electric or magnetic dipoles for some relevant quantum technologies considered in the text, along with their energy transitions (see the summary in Table A1). We are using the following assumptions: (a) transitions occur between n→n−1 (adjacent level), *n* being the thermal occupancy; and (b) the approximate radial electric dipole momentum becomes d∼$n^{2}ea_{0}$ with *e* being the elementary charge and $a_{0}$ the Bohr radius.

### Appendix B.1. QuEra (Rydberg Atoms)

For transitions between Rydberg states in Rubidium-87 (^87^Rb) [60], considering f=100GHz (the approximate frequency generated on the atom due to the interaction with the radiation field) and n=50 (the average principal number of common transitions in these atoms considered for the expected orbital radius), we have the following.

$$\begin{array}{ccc} f & = & 1.00\times 10^{11}\;\mathrm{Hz}\\ \omega =2\pi f & = & 6.28\times 10^{11}\;s^{-1}\\ \mu ∼n^{2}a_{0}e & = & 2.12\times 10^{-26}\;C\cdot m \end{array}$$

### Appendix B.2. QED Optical Transitions

For QED optical transitions from the fundamental state, where the atoms have wavelengths of λ=600nm, we have:

$$\begin{array}{ccc} f=\frac{c}{\lambda } & = & 5.00\times 10^{9}\;\mathrm{Hz}\\ \omega =2\pi f & = & 3.14\times 10^{15}\;s^{-1}\\ \mu ∼ea_{0} & = & 8.48\times 10^{-30}\;C\cdot m \end{array}$$

### Appendix B.3. Nitrogen Vacancy Centres

For NV centres, considering magnetic dipole transitions for the fundamental state, we have:

$$\begin{array}{ccc} f & = & 2.87\times 10^{9}\;\mathrm{Hz}\\ \omega =2\pi f & = & 1.80\times 10^{10}\;s^{-1}\\ \mu =\mu_{B}=\frac{e\hbar }{2m_{e}} & = & 9.27\times 10^{-24}\;A\cdot m^{2}\;\left(\mathrm{Bohr}\mathrm{magneton}\right) \end{array}$$

**Table A1:** Summary of estimations of electric or magnetic dipoles for selected quantum technologies, with their energy transitions.

Technology	f(Hz)	$\omega \;\left(s^{-1}\right)$	Dipole Momentum
Rydberg atoms	$1.00\times 10^{11}$	$6.28\times 10^{11}$	$2.12\times 10^{-26}\;C\cdot m$
Optical transitions	$5.00\times 10^{9}$	$3.14\times 10^{15}$	$8.47\times 10^{-30}\;C\cdot m$
NV Centres	$2.87\times 10^{9}$	$1.80\times 10^{10}$	$9.27\times 10^{-24}\;A\cdot m^{2}$
